# *SERPINB3*, Adult-Onset Immunodeficiency, and Generalized Pustular Psoriasis

**DOI:** 10.3390/genes14020266

**Published:** 2023-01-19

**Authors:** Piranit Kantaputra, Teerada Daroontum, Mati Chuamanochan, Suteeraporn Chaowattanapanit, Salin Kiratikanon, Charoen Choonhakarn, Worrachet Intachai, Bjorn Olsen, Sissades Tongsima, Chumpol Ngamphiw, Patrizia Pontisso, Timothy C. Cox, Puey Ounjai

**Affiliations:** 1 Center of Excellence in Medical Genetics Research, Faculty of Dentistry, Chiang Mai University, Chiang Mai 50200, Thailand; 2 Division of Pediatric Dentistry, Department of Orthodontics and Pediatric Dentistry, Faculty of Dentistry, Chiang Mai University, Chiang Mai 50200, Thailand; 3 Department of Pathology, Faculty of Medicine, Chiang Mai University, Chiang Mai 50200, Thailand; 4 Division of Dermatology, Department of Internal Medicine, Faculty of Medicine, Chiang Mai University, Chiang Mai 50200, Thailand; 5 Division of Dermatology, Department of Medicine, Faculty of Medicine, Khon Kaen University, Khon Kaen 40002, Thailand; 6 Department of Developmental Biology, Harvard School of Dental Medicine, Harvard University, Boston, MA 02115, USA; 7 National Biobank of Thailand, National Science and Technology Development Agency (NSTDA), Thailand Science Park, Pathum Thani 12120, Thailand; 8 Department of Medicine, University of Padua, 35128 Padua, Italy; 9 European Reference Network—ERN RARE-LIVER, 72076 Tübingen, Germany; 10 Departments of Oral & Craniofacial Sciences, School of Dentistry, and Pediatrics, School of Medicine, University of Missouri-Kansas City, Kansas City, MO 64108, USA; 11 Center of Excellence on Environmental Health and Toxicology (EHT), Office of Higher Education Commission, Ministry of Education, Bangkok 10400, Thailand; 12 Department of Biology, Faculty of Science, Mahidol University, Bangkok 10400, Thailand

**Keywords:** adult-onset immunodeficiency syndrome, anti-interferon-γ autoantibody, hyperactive elastase activity, SERPIN, *SERPINB3* mutation, generalized pustular psoriasis, pustular skin reaction

## Abstract

Background: Generalized pustular psoriasis (GPP; MIM 614204) is a rare and severe pustular autoinflammatory skin disease in which acute generalized erythema and scaling develop with numerous sterile pustules. GPP shares skin manifestations, especially pustular skin reaction, with adult-onset immunodeficiency (AOID) with anti-interferon-γ autoantibodies, an autoimmune disease. Methods: Clinical examinations and whole-exome sequencing (WES) were performed on 32 patients with pustular psoriasis phenotypes and 21 patients with AOID with pustular skin reaction. Immunohistochemical and histopathological studies were performed. Results: WES identified three Thai patients presenting with similar pustular phenotypes—two with a diagnosis of AOID and the other with GPP. A heterozygous missense variant chr18:g.61325778C>A NM_006919.2: c.438G>T; NP_008850.1: p.Lys146Asn; rs193238900 in *SERPINB3* was identified in two patients: one with GPP and the other with AOID. The other patient who had AOID carried a heterozygous missense variant chr18:g.61323147T>C NM_006919.2: c.917A>G; NP_008850.1: p.Asp306Gly in *SERPINB3*. Immunohistochemical studies showed overexpression of SERPINA1 and SERPINB3, a hallmark of psoriatic skin lesions. Conclusions: Genetic variants in *SERPINB3* are associated with GPP and AOID with pustular skin reaction. The skin of patients with GPP and AOID carrying *SERPINB3* mutations showed overexpression of SERPINB3 and SERPINA1. Clinically and genetically, GPP and AOID appear to share pathogenetic mechanisms.

## 1. Introduction

Generalized pustular psoriasis (GPP; MIM 614204) is a rare and severe pustular autoinflammatory skin disease in which acute generalized erythema and scaling develop with numerous aseptic pustules. GPP shares skin manifestations, especially pustular skin reaction, with adult-onset immunodeficiency (AOID) with anti-interferon (IFN)-γ autoantibodies, an autoimmune disease [1]. Patients with AOID have symptoms of AIDS-like illness with disruptive IFN-γ signaling, including recurrent and disseminated opportunistic infections, especially non-tuberculous mycobacteria and varicella zoster virus. The clinical manifestations of patients with AOID resemble those in patients with inborn deficiencies of IFN-γ response or IFN-γ- production. Patients with AOID may have pustular skin reactions similar to those found in patients with GPP [1,2].

Genetic variants in *IL36RN*, *IL1RN*, *CARD14*, *AP1S3, MPO,* and *TNIP1* have been shown to be implicated in GPP [1,2,3]. Very recently, genetic variants in *SERPINA1* (Serine peptidase inhibitor, clade A, member 1; MIM 107400) [3] and *SERPINA3* (Serine peptidase inhibitor, clade A, member 3; MIM 107280) [4] have been reported as predisposing risk factors for GPP as well as AOID [3,5], indicating that variants in serine peptidase inhibitors are implicated in pathogenetic mechanisms of both disorders.

The SERPIN protein superfamily represents the largest group of proteases. Although most are highly effective inhibitors of serine proteases, some members also, or even exclusively, only inhibit cysteine proteases. SERPINS are expressed in a diverse array of tissues and control many biological responses, including apoptosis and inflammation [6,7]. Given the implication of a role for *SERPINA1* and *SERPINA3* variants in the pathogenetic mechanisms of GPP and AOID, we hypothesized that genetic variants in other members of the SERPIN superfamily, particularly those highly expressed in skin and/or the immune system, might also predispose patients to GPP and AOID. In this regard, SERPINB3 (clade B member; MIM 600517) represented an ideal candidate, as *SERPINB3* is expressed in normal epithelial cells and overexpressed in various damaged cell types, including hepatocytes and cancer cells [8]. Regarding skin, *SERPINB3* is expressed in the spinous and granular layers of the normal epithelium and has an important role in regulating differentiation of the squamous epithelium [9]. In addition, *SERPINB3* is highly expressed in psoriatic skin because it is induced by pro-inflammatory cytokines secreted by infiltrated immune cells. Here, its expression helps prevent apoptosis. It is also found to be overexpressed in neoplastic tissue of epithelial origin [9,10,11]. The roles of *SERPINB3* are to regulate the immune system, proteolysis homeostasis, and apoptosis. Patients with *SERPINB3* dysfunction are known to be prone to altered immune responses and autoantibody production [12]. In addition, the over-accumulation of abnormal SERPINB3 might increase the levels of Pso p27, an autoantigen derived from SERPINB3 and SERPINB4, which is highly involved in the inflammatory process in psoriasis [13].

To provide insight into the genetic architecture of pustular skin reactions, we undertook whole-exome sequencing of 53 patients of Thai descent that presented with either GPP or AOID. We describe herein the identification of rare genetic variants in *SERPINB3* in three unrelated Thai patients: two with AOID and a pustular skin reaction and the third with GPP. One AOID patient and a patient with GPP carried the same heterozygous missense variant in *SERPINB3*. The other AOID patient had a novel heterozygous missense variant in *SERPINB3*. We further show that both SERPINA1 and SERPINB3 are highly expressed in the pustular skin biopsies of patients with *SERPINB3* mutations. This is the first report suggesting that genetic variants in *SERPINB3* predispose patients to various pustular skin reactions and further support the notion that GPP and AOID share pathogenetic pathways.

## 2. Materials and Methods

### 2.1. Patients

This study involving human participants was approved by the Human Experimentation Committees of the Faculty of Medicine, Chiang Mai University, the Faculty of Dentistry, Chiang Mai University (no. 71/2020), and the Faculty of Medicine, Khon Khan University and was performed in accordance with the ethical standards of the 1964 Declaration of Helsinki and its later amendments or comparable ethical standards. Informed consent was obtained from all participants.

Clinical examinations and whole-exome sequencing were performed on our cohort of 53 patients with pustular skin reaction, including 21 patients with AOID and 32 patients with pustular psoriasis phenotypes. The inclusion criteria were patients with AOID or pustular psoriasis phenotypes, including GPP, PPP, and ACH. The exclusion criteria were patients without AOID or pustular psoriasis phenotypes. The clinical and molecular findings of the patients are summarized in Table 1.

#### 2.1.1. Patient 1

A 28-year-old woman presented with persistent generalized multiple sterile pustular lesions without systemic symptoms. A diagnosis of GPP was made. The pustular rash responded well to oral acitretin leading to complete remission in 2 years. At age 36 years, she developed generalized well-defined scaly erythematous plaques without pustulation compatible with plaque-type psoriasis, which was well-controlled with oral acitretin. No nail or joint involvements during the clinical course were noted.

#### 2.1.2. Patient 2

A 37-year-old Thai female presented with a 5-month history of a low-grade fever with generalized lymphadenopathy and weight loss (10 kg in 3 months). A cervical lymph node biopsy was performed for histopathology, mycobacterial and fungal cultures, and PCR. The histopathology was compatible with chronic lymphadenitis. The mycobacterial culture and PCR showed *Mycobacterium abscessus*. The anti-HIV test was negative, but the anti-interferon-γ autoantibodies test was positive at 1:400,000. While waiting for the results, the patient developed a generalized pustular eruption. The Gram stain from the pustules revealed no organisms, and the bacterial culture was negative. The patient was diagnosed with disseminated *M. abscessus* infection with adult-onset immunodeficiency due to the anti-interferon-γ autoantibodies with pustular skin eruption. Anti-mycobacterial therapy with azithromycin (500 mg/day) and ciprofloxacin (1000 mg/day), and prednisolone (60 mg/day with a taper dose by 10 mg every two weeks) was initiated. Her symptoms, including generalized lymphadenopathy and pustular eruption, significantly improved after the treatment. However, generalized lymphadenopathy and pustular eruption flared up from time to time, and oral acitretin was added to control the pustular eruption.

#### 2.1.3. Patient 3

A 60-year-old Thai male presented with a 3-month history of a low-grade fever with chronic nonproductive cough, generalized lymphadenopathy, and weight loss (7 kg in 3 months). Two weeks before admission to Srinagarind Hospital, the patient developed multiple discrete erythematous papules and pustules on both extremities. A cervical lymph node biopsy was performed. The histopathological result was consistent with reactive paracortical hyperplasia. The tissue cultures for mycobacteria and fungus were negative. The PCR test was positive for nontuberculous mycobacteria, but the species could not be identified. A skin biopsy was performed, and the histopathological result was consistent with subcorneal spongioform pustulosis. The anti-HIV test was negative, but the anti-interferon-γ autoantibodies test was positive at 1:200,000. The patient was diagnosed with disseminated nontuberculous mycobacteria infection with adult-onset immunodeficiency due to the anti-interferon-γ autoantibodies with pustular eruption. Anti-mycobacterial therapy with clarithromycin (1000 mg/day) and moxifloxacin (400 mg/day), and prednisolone (30 mg/day with a decreasing dose of 10 mg every two weeks) was started. The patient’s symptoms and skin lesions improved after the treatment; however, the patient developed a flare-up of disease and pustular lesions. Acitretin was added to control the lesions.

### 2.2. Whole-Exome Sequencing and Mutation Analysis

All the consented patients’ genomic DNA were extracted from whole blood with an automated DNA-extracting machine, Autogen QuickGene-810 (FUJIFILM Corporation, Tokyo, Japan). The DNA was sent to Macrogen, Korea to perform whole-exome sequencing (WES). This WES service used Agilent SureSelect^XT2^ Human All Exon V6 + UTR target enrichment (PR7000-0152; Agilent Technologies, Santa Clara, CA, USA) to capture exonic regions to be sequenced. The sequencing reads in FASTQ format were fed to the Genomics analysis toolkit (GATK) germline mutation workflow version 3.8.1 [14,15] to identify the variants. These input sequencing reads were aligned to the human genome reference (hg19) using BWA-MEM version 0.7.17 [14,15] to generate BAM files. The BAM files were then processed using GATK HaplotypeCaller to identify SNVs and small indels resulting in individual GVCF files. These GVCF files were consolidated into a single-joint genotyped VCF file format listing all genotypes in separate columns.

The Ensemble variant effect predictor tool (version 95) [16] was used to predict the pathogenic effects of each variant. Standard variant filtering pipelines, based on allele frequency, CADD scores (>15), and pathogenicity algorithms, were applied to identify rare variants of potential interest. The mutation predictions were performed by using MutationTaster (https://www.mutationtaster.org) (accessed on 10 January 2023), PolyPhen-2 (http://genetics.bwh.harvard.edu/pph2) (accessed on 10 January 2023), and SIFT (https://sift.bii.a-star.edu.sg) (accessed on 10 January 2023). All of the variants identified with WES were confirmed with Sanger sequencing.

### 2.3. Histopathology

The skin lesions of patients 2 and 3 were biopsied and then instantly fixed in 10% neutral-buffered formalin solution. The tissues were embedded in the paraffin block after fixing, dehydrating, clearing, and infiltrating paraffin wax. The paraffin-embedded tissues were sectioned at a thickness of 3 µm for histopathological analysis, and they were then stained with hematoxylin and eosin (H&E) according to the standard histological laboratory methods. The histopathologic characterization of the specimens was evaluated by experienced dermatologists and dermatopathologists.

### 2.4. Immunohistochemistry

Sections of 3 µm-thick, formalin-fixed, paraffin-embedded tissue were cut and placed on Superfrost Plus microscope slides. The slides were heated for one hour at 60 °C in a dry oven to enhance tissue attachment and soften the paraffin. The immunohistochemistry procedure was performed on a Ventana BenchMark ULTRA autostainer using the standard protocol. In summary, the sections underwent deparaffinization, rehydration, and antigen retrieval using CC1 (prediluted, PH 8.0) antigen retrieval solution (Ventana). The primary antibodies were added and incubated with the sections at a dilution suggested by the manufacturer. The following primary antibodies were used: mouse monoclonal anti-human SerpinB3/SCCA [clone OTI1A5] (ab180396, Abcam, Cambridge, MA, USA, 1:100 dilution) and rabbit monoclonal anti-human α 1 Antitrypsin (Serpina1) [clone EPR17087-50] (ab207303, Abcam, Cambridge, MA, USA, 1:400 dilution). The visualization process was performed using the Ultraview universal DAB IHC detection kit, and it was afterwards counterstained using hematoxylin and bluing solution. The slides were gently cleaned, dehydrated in graded ethanol and xylene, and then mounted using mounting media on a microscope slide.

### 2.5. Molecular Modeling of the SERPINB3 Variants

The amino acid sequence of the *SERPINB3* (NP_008850.1) was obtained from the NCBI GenBank Database. The 3D models of both the wild type and missense mutation NP_008850.1:p.Lys146Asn variant were constructed using the *SERPINB4* structure (PDB ID: 4zk0.1 [17]) as a template. The NP_008850.1: p.Asp306Gly variant was constructed using the crystal structure of a domain-swapped serpin dimer (PDB ID:2ZNH [18] as a template. The molecular modeling was done using the SWISS-MODEL server [19]. Further structural evaluation of the molecular models of both the wild type and mutant proteins was conducted using MolProbity [20]. The structural visualization was obtained using the UCSF Chimera software package [21].

## 3. Results

### 3.1. Whole-Exome Sequence Sequencing and Bioinformatic Analysis

**Figure 1:**
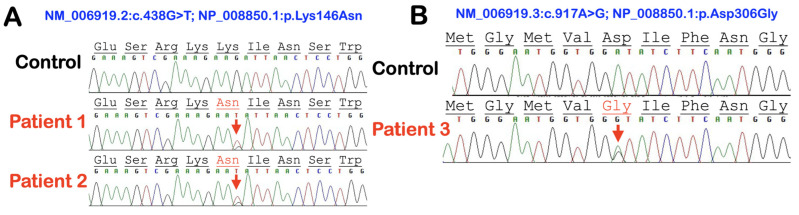
Electropherograms of *SERPINB3* variants and a normal control. (**A**) The heterozygous base substitution c.438G>T in patients 1 and 2 is predicted to cause the heterozygous missense variant p.Lys146Asn. (**B**) The heterozygous base substitution c.917A>G in patient 3 is predicted to cause the heterozygous missense variant p.Asp306Gly.

**Figure 2:**
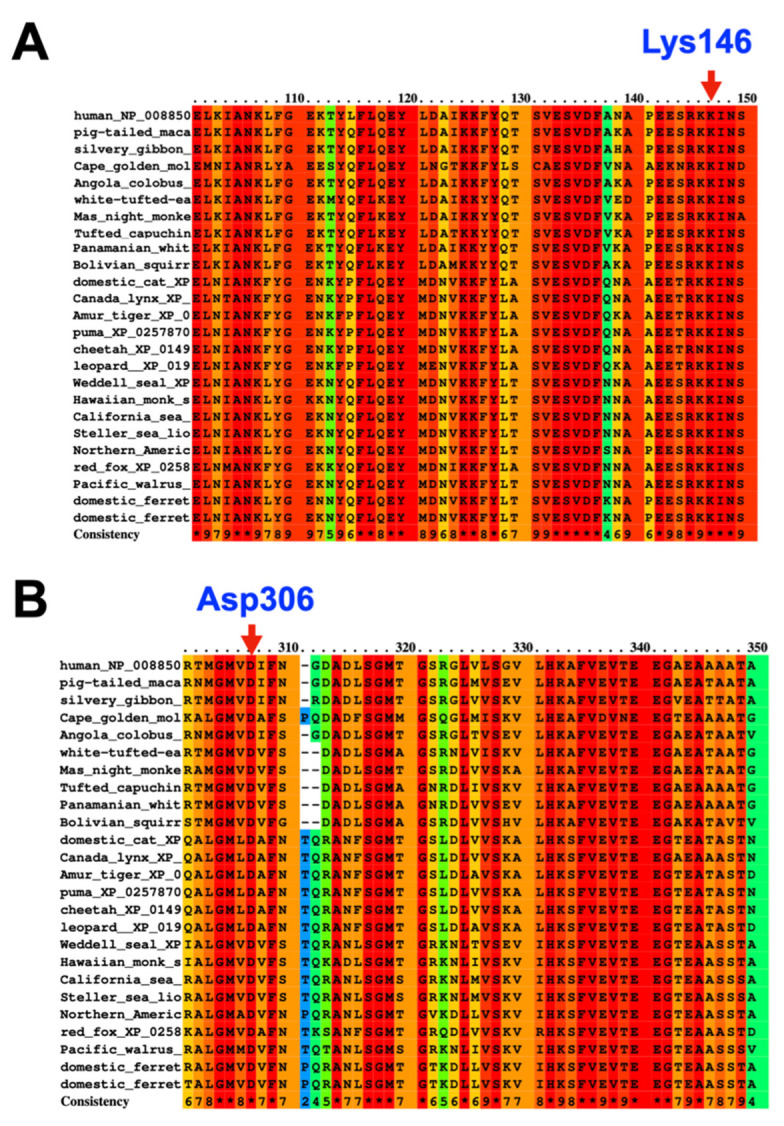
Conservation of SERPINB3 amino acids. The amino acid residues (**A**) Lys146 and (**B**) Asp306 are highly conserved across species.

In our cohort, we identified three extremely rare deleterious variants in *SERPINB3* whose allele frequencies were less than 0.0000999 according to gnomAD v2.1.1 (https://gnomad.broadinstitute.org) (accessed on 10 January 2023). A heterozygous missense variant chr18: g.61325778C>A NM_006919.2: c.438G>T; NP_008850.1: p.Lys146Asn; rs193238900 in *SERPINB3* was identified in patients 1 and 2. The Lys146 residue is highly conserved (Figure 1A and Figure 2A). The p.Lys146Asn mutation is predicted to be disease causing (0.01) and probably damaging (0.991) with MutationTaster and PolyPhen-2, respectively (Table 1).

A heterozygous missense variant chr18: g.61323147T>C NM_006919.2: c.917A>G; NP_008850.1: p.Asp306Gly in *SERPINB3* was identified in patient 3 (Figure 1B and Figure 2B). The p.Asp306Gly mutation is predicted to be deleterious (0) and possibly damaging (0.889) with MutationTaster and PolyPhen-2, respectively (Table 1). In addition to these *SERPINB3* variants, variants in other immunodeficiency genes, including *SERPINA5*, *SERPINA9*, *IL1RN*, and *IL1RL2*, were also identified in patients 2 and 3 (Table 1).

### 3.2. Histopathological Findings

**Figure 3:**
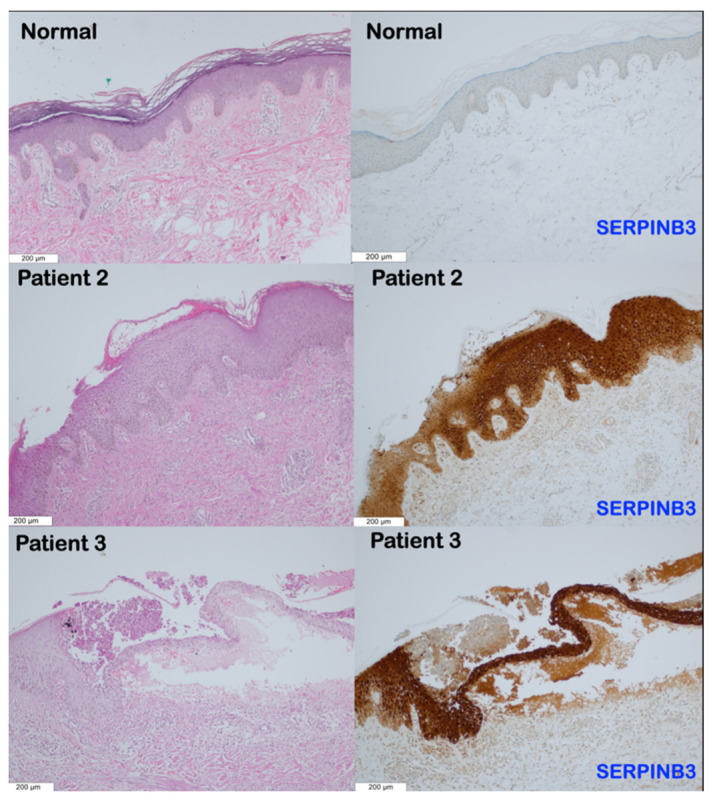
Representative immunohistochemistry images for SERPINB3 in the normal skin control and patients 2 and 3. The epidermis of the normal skin showed very low levels of SERPINB3 expression, while the dermis lacked expression. In comparison to the normal skin tissue, there was a noticeably higher level of SERPINB3 expression in the stratum basale, stratum spinosum, stratum granulosum including the subcorneal pustule, and upper dermis of patients 2 and 3. (Magnification 10×).

**Figure 4:**
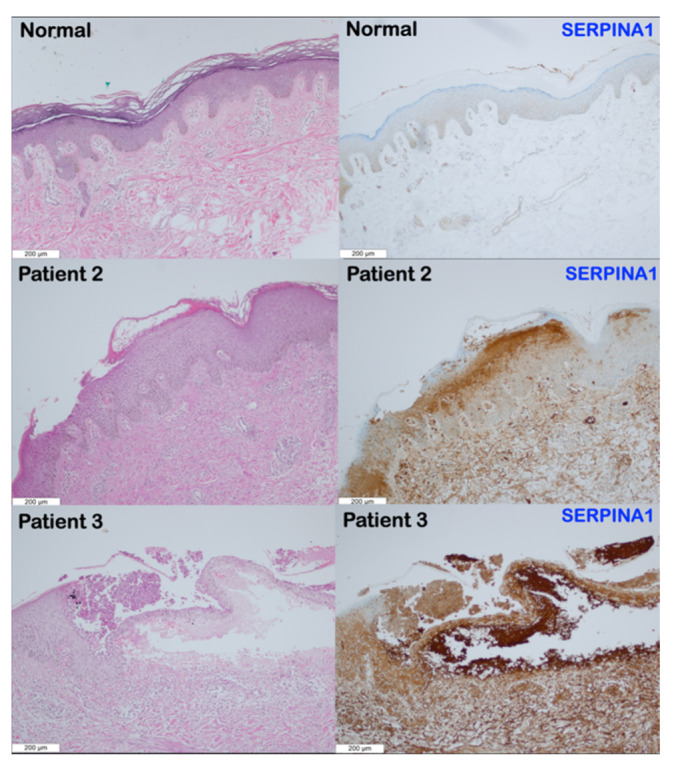
Representative immunohistochemistry images for SERPINA1 in the normal skin control and patients 2 and 3. The normal skin expresses SERPINA1 at low levels in the epidermis; no expression is seen in the dermis. SERPINA1 expression is significantly higher in the subcorneal pustule, stratum granulosum, stratum spinosum, stratum basale, and upper dermis of patients 2 and 3 than in the normal skin. (Magnification 10×).

The histological analyses of the skin biopsies from patients 2 and 3 revealed remarkable pathology. In patient 2 with AOID, lesional skin histology revealed subcorneal neutrophilic accumulation with epidermal acanthosis, spongiosis, and parakeratosis. The upper dermis showed perivascular lymphocytic infiltration (Figure 3 and Figure 4). In patient 3 with AOID, lesional skin histology showed subcorneal and intraepidermal pustules with significant neutrophils collections. The adjacent epidermis displayed parakeratosis, spongiosis, and acanthosis. Dermal–epidermal separation was also observed. The upper dermis showed perivascular lymphocyte and few neutrophil infiltrations (Figure 3 and Figure 4).

### 3.3. Immunohistochemical Findings

Immunohistochemical studies showed increased expression of *SERPINB3* and *SERPINA1* in the subcorneal pustule, stratum granulosum, stratum spinosum, stratum basale, and upper dermis of patients 2 and 3 compared to the normal skin tissue (Figure 3 and Figure 4).

## 4. Discussion

### 4.1. SERPINB3 Variants and GPP and AOID Phenotypes

**Figure 5:**
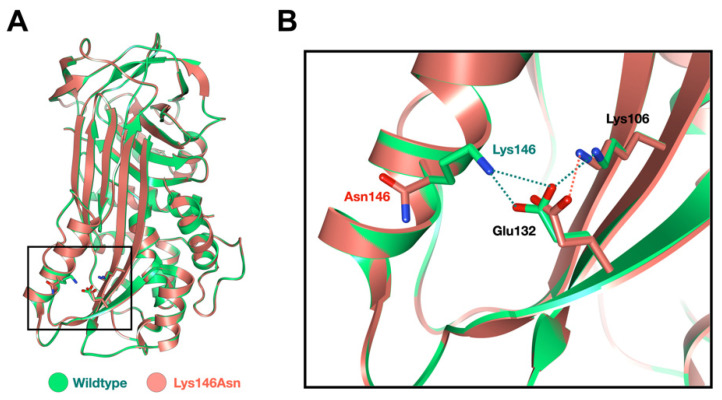
The point mutation at Lys146 alters the salt bridge formation between helix-F and β-sheet A. (**A**) Superimposition of the homology-based 3D models of the SERPINB3 and its p.Lys146Asn variant (K146N) showing the position of amino acid residue-146 and its inter-reacting partners. The normal SERPINB3 is colored in green, and the p.Lys146Asn variant is colored in salmon. (**B**) The expanded view of the selected area in A revealed the network of salt bridges between Lys146 and highly conserved Glu132 and Lys106. The disruption of this interaction network caused by the Asn substitution could significantly alter the stability of the SERPIN structure.

In this study, we report three unrelated Thai patients: two with AOID and the third with GPP. Patients 1 and 2 who had AOID with pustular reaction and GPP, respectively, carried the same heterozygous missense variant c.438G>T; p.Lys146Asn in *SERPINB3,* suggesting shared pathogenetic pathways between GPP and AOID. Patient 3 who was affected with AOID carried a novel heterozygous missense variant c.917A>G; p.Asp306Gly in *SERPINB3*. Our findings raise an interesting question of whether both *SERPINB3* variants were implicated in GPP and AOID phenotypes or were just coincidental findings. We have reason to believe that these variants are implicated in GPP and AOID. First, *SERPINB3*, *SERPINA1,* and *SERPINA3* are homologous and share inhibitory function, and genetic variants in *SERPINA1 and SERPINA3* have been reported to be the “predisposing risk factors” for GPP [4,5] and AOID [3]. Second, the c.438G>T; p.Lys146Asn variant found in the two unrelated patients is very rare in the general population. According to gnomAD, its allele frequency is 0.0002126, and it is not seen in 30598 alleles in the South Asian population. It was detected in two of 720 Thai individuals who did not have a history of pustular reaction in our in-house exome database (allele frequency = 0.00069444). Third, the amino acid p.Lys146 residue is highly conserved (Figure 2A). The in silico model of the p.Lys146Asn variant revealed that the substitution of Lys146 with Asn altered the formation of the salt bridge between helix F and β-sheet A. The cluster of interacting residues in both helix F and β-sheet A was proposed to be significant for maintaining structural stability and thus increases resistance in polymerization [22,23]. Notably, the mutation in helix F appeared to accelerate the polymerization process in certain SERPIN proteins [24] (Figure 5). This mutation is predicted to be deleterious (0.01) and probably damaging (0.991) with SIFT and PolyPhen, respectively (Table 1).

The c.917A>G; NP_008850.1:p.Asp306Gly variant in *SERPINB3*, which was identified in patient 3 affected with AOID, appears to be novel. It was not reported in the gnomAD and LOVD databases. It was not found in the 720 Thai individuals of our in-house exome database. The mutation is predicted to be deleterious (0) and possibly damaging (0.889), with SIFT and PolyPhen, respectively (Table 1).

**Figure 6:**
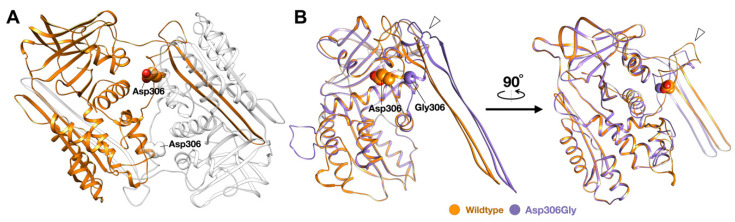
Molecular model of dimeric SERPINB3. (**A**) The ribbon diagram of the modelled polymeric serpin reveals the position of conserved residue Asp306 in the loop region of the proteins. (**B**) The superimposed structures of the wildtype and Asp306Gly variant show significant structural alteration in the loop region of SERPINB3, implying that glycine substitution at this position could potentially affect the conformational flexibility of the loop region (white arrow heads), which may promote polymerization of the variant proteins.

The amino acid Asp306 residue is highly conserved (Figure 2B). The in silico protein modeling of the dimer structure of *SERPINB3* proteins shows that the amino acid Asp306 residue is located at a flexible loop region. The glycine substitution in this loop region could cause an increase in the overall structural flexibility of the loop area. This may lead to an increase in conformational lability and formation of the M* state, resulting in a possible rise in SERPIN polymerization [18] (Figure 6).

### 4.2. SERPINB3 Variant Predisposed Patients to GPP

**Figure 7:**
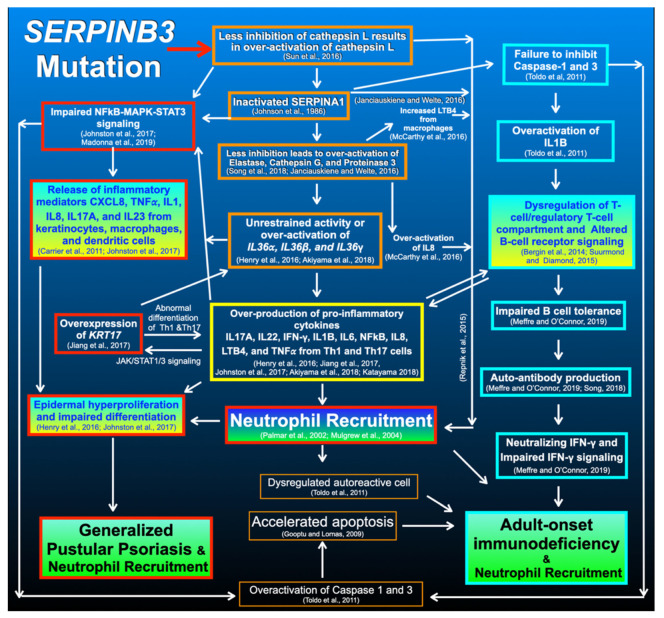
Hypothetical flowchart of the pathogenetic pathways that result from *SERPINB3* mutation. Mutations in *SERPINA1* and *SERPINB3* share “over-activation effects of elastase”, which result in unrestrained activities of IL36 cytokines and the subsequent “domino effects” that lead to GPP and AOID with neutrophil recruitment at the end. Refs. [25,28,29,30,31,32,33,34,35,36,37,38,39,40,41,42,43,44,45,46].

The roles of *SERPINB3* are to regulate the immune system, modulate proteolysis homeostasis, and attenuate physiologic apoptosis [25] in order to defend against the invasion of microorganisms and prevent self-tissue destruction as a result of excessively unrestrained proteolysis. Our findings raise the question of how a genetic variant in *SERPINB3* predisposed the patient to GPP. *SERPINB3* functions by inhibiting cysteine protease cathepsins L, S, and K, and subsequently suppresses inflammatory reactions [25,26] (Figure 7). This is supported by the finding of an excessive pro-inflammatory response in *Serpinb3a* knockout mice [27].

The main function of SERPINB3 is to suppress inflammation by inhibiting cathepsin L [25], and cathepsin L inactivates SERPINA1 (α-1-antiprotease inhibitor) [28], which subsequently inhibits the downstream elastase [29], the key enzyme that activates IL36 cytokines (Figure 7) [30,31]. Mutations in *SERPINA1* are implicated in autoinflammatory diseases, including emphysema, liver cirrhosis, and skin panniculitis, as a result of over-activation of elastase and accumulation of mutant polymers [32]. In addition, very recently, we reported a rare variant in *SERPINA1* in patients with GPP and AOID [5]. Therefore, it is hypothesized that the aberrant expression of *SERPINB3* as a result of its mutation may result in protein misfolding and polymerization leading to endoplasmic reticulum stress; overproduction of cathepsin L; impaired SERPINA1 function; overactivation of elastase; unrestrained activity of IL36 cytokines; over-production of pro-inflammatory cytokines, including IFN-γ, IL-17, IL-22, IL1B, IL6, IL8, LTB4, TNF-α, and TGF-β; overexpression of *KRT17*; hyperactive NFkB-MAPK-STAT3 signaling; impaired epidermal differentiation; epidermal hyperproliferation; and subsequent GPP with neutrophil recruitment (Figure 7) [29,31,33].

In addition to the effects of an elevated expression of *IL8, IL6*, and *LTB4* as a result of the *SERPINB3* variant, neutrophil recruitment in patients with the *SERPINB3* variant may be the result of inactivated SERPINA1 [34] and overproduction of cathepsin L because cathepsin L via chemokine processing produces active forms of angiogenic ELR-CXC chemokines that result in neutrophil recruitment (Figure 7) [47].

### 4.3. SERPINB3 Variants Predispose Patients to AOID

The next question is what did the *SERPINB3* mutations do to predispose the patients to the autoimmune disease AOID with the production of IFN-γ autoantibodies and pustular reaction. Autoimmune responses in patients with *SERPINB3* mutations may be the result of endoplasmic reticulum stress secondary to the accumulation of polymers of misfolded proteins [48] and maldevelopment of B cells because *SERPINB3* is expressed in CD27^+^ B cells and plays an important role in normal B cell activation [49]. In addition, overactivation of cathepsin L as a result of *SERPINB3* mutations may lead to dysregulated antigen presentation because cathepsin L has an important role in priming adaptive immune cells [50] and negatively regulating B lymphocyte production [51] (Figure 7).

Of note, apoptosis is central to regulating the immune system by involving the maintenance of self-tolerance and homeostatic control of lymphocyte populations. SERPINB3 plays crucial roles in attenuating apoptosis [25] by its inhibitory effect via the JNK-p38 signaling pathway [52], its protective effect against lysosomal injury [53], contrasting cytochrome c release from the mitochondria [54], and chemotactic effect for natural killer cells [35]. Dysregulated apoptosis as a result of aberrant *SERPINB3* expression has been shown to be implicated in autoimmune diseases such as systemic lupus erythematosus because the inefficient disposal of apoptotic debris results in the rescue of autoreactive immune cells [12].

Therefore, aberrant *SERPINB3* expression as a result of *SERPINB3* mutations may result in the accumulation of polymers of misfolded SERPINB3 proteins, overactivation of cathepsin L, inactivation of *SERPINA1*, dysregulated B cell reactivity, impaired immune homeostasis, disruptive apoptotic mechanism, increased autoantigen burden, dysregulated autoreactive cell proliferation, and subsequently an autoimmune disorder such as AOID with pustular reaction [35,55] (Figure 7).

### 4.4. Overexpression of SERPINA1 and SERPINB3 in Patients with SERPINB3 Variants

The reason why SERPINA1 and SERPINB3 are overexpressed in the lesional skin biopsy of patients with a *SERPINB3* variant is unknown. It might be due to the increased inflammation/oxidative stress as a result of the mutations, in turn increasing the expression of these serpins, including SERPINB3 and SERPINA1, in a positive loop.

### 4.5. Overproduction of IFN-γ Autoantibodies in Patients with AOID

The production of IFN-γ autoantibodies in patients with AOID may be the result of *SERPINB3* mutation-associated aberrant *SERPINA1* expression because *SERPINA1* regulates neutrophil-driven autoantibodies via TNF-α intracellular signaling and neutrophil degranulation of tertiary and secondary granules [36]. Pro-inflammatory cytokines and overactivation of NFκB signaling may result in dysregulation of T cell homeostasis, B cell tolerance defects, and subsequent impaired counter-select developing autoreactive B cells thus stimulating the development of autoimmunity and the clinical manifestations of AOID through the presentation of self-antigens to T cells [37].

Our study supports the biological roles of *SERPINB3* in inhibiting cysteine proteases when they are mutated, and the overactivation of proteases leads to an excessive inflammatory reaction with neutrophil recruitment. This study suggests that GPP and AOID with pustular reaction are diseases that result from dysregulated proteolytic and apoptotic pathways. The shared genetic vulnerability for GPP and AOID, mediated by *SERPINB3* variants, implicates these variants as “predisposing risk factors” for GPP and AOID with pustular reaction. Collectively, it appears that mutations in *SERPINA1* and *SERPINB3* share “over-activation effects of elastase”, which result in unrestrained activities of IL36 cytokines and subsequent “domino effects” leading to GPP and AOID with neutrophil recruitment at the end (Figure 7).

The complex balance of the protease/anti-protease activity should, however, also take into account the fact that an excess of anti-protease activity, as described in the *SERPINB3* variant SCCA-PD (NP_008850.1:p.Gly351Ala) [55,56] seen in 24% of the normal population, could alter cell homeostasis, leading in this case to an increased pro-fibrogenic profile and more severe liver portal hypertension in patients with cirrhosis (Martini et al. manuscript in preparation). This genetic predisposition, as it occurs in the above-mentioned mutations in the presence of different hits, could lead to clinical manifestations and supports the need for personalized approaches, not only in patient clinical management but also in preventive medicine strategies.

### 4.6. SERPINB3 Mutations, Pso p27, and Pustular Skin Reaction

*SERPINB3* and *SERPINB4* are known to be overexpressed in epidermal cells of psoriatic skin [11]. The serpin-derived protein Pso p27, an autoantigen in psoriasis and other chronic inflammatory diseases, is proteolytically derived from SERPINB3 and SERPINB4 through non-canonical cleavage in mast cells by chymase [13]. In psoriasis, Pso p27 is a major locally generated immunogen, which is exclusively expressed in psoriatic skin lesions but not in the normal skin [13,57]. The immune complex formed by Pso p27 and corresponding antibodies activates the complement system and aggravates the inflammatory process. Pso p27 plays roles in the autoimmune reaction in psoriasis [13,58] and, possibly, in AOID with pustular skin reaction as well. The amount of Pso p27 is correlated to disease activity. Therefore, mutations in *SERPINB3* in our patients might result in an overexpression not only of SERPINB3 (Figure 4) but also of Pso p27, and this could profoundly aggravate skin inflammation with neutrophil infiltration.

The genetic variants in other immunodeficiency genes, including *SERPINA5, SERPINA9, IL1RN*, and *IL1RL2*, might aggravate the inflammatory process in patients 2 and 3 (Table 1). In this regard, a shared variant in *SERPINA9* (*Serpin peptidase inhibitor*, *cladeA*, *member 9*; MIM 615677) was identified in both AOID patients (patients 2 and 3). The variant in SERPINA9, an inhibitor of trypsin and trypsin-like serine proteases, might contribute to the pathogenesis of AOID because *SERPINA9* has important roles in the development and function of B cells [59]. Therefore, alteration of *SERPINA9* might contribute to the altered autoimmune responses in patients along with the variants in *SERPINB3*. Patient 1 with GPP and patient 2 with AOID carrying the same *SERPINB3* variant (p.Lys146Asn) suggests shared pathogenetic mechanisms of GPP and AOID. Our paper will encourage dermatologists and geneticists to screen other SERPIN genes for “predisposing risk factors” for GPP and AOID in patients who do not carry mutations in the other known genes for GPP and AOID.

## 5. Conclusions

In conclusion, we report for the first time that genetic variants in *SERPINB3* might predispose patients to GPP and AOID with pustular skin reaction. Interestingly, SERPINA1 and SERPINB3 are overexpressed in the pustular skin of patients with *SERPINB3* mutations. We also present hypothetical pathogenetic pathways leading to GPP and AOID with pustular skin reaction as a result of *SERPINB3* mutations and subsequent overactivation of cathepsin L. All lines of evidence imply that both GPP and AOID share pathogenetic mechanisms. Our findings suggest that antibodies to SERPINA1 and SERPINB3 might be used to treat GPP and AOID with pustular skin reaction.

## Figures and Tables

**Table 1:** Patients with *SERPINB3* variants and variants in other immunodeficiency genes.

Patients	Dx	*SERPINB3* Variants	*SERPINA5* Variant	*SERPINA9* Variant	*IL1RN* Variant	*IL1RL2* Variant
**1**	GPP	chr18: g.61325778C>A NM_006919.2: c.438G>T NP_008850.1 p.Lys146Asn rs193238900 Allele freq = 0.0002126				
**2**	AOID			chr14:g.94929551G>A NM_001042518.2 c.833C>T NP_001035983.2 p.Ser278Leu rs370040760 Allele freq = 0.00006406	NM_000577.5 c.392C>T NP_000568.1 p.Ala131Val rs374276440 Allele freq = 0.00003183	NM_003854.2 c.791G>A NP_003845.2 p.Arg264Lys Allele freq = 0.0001875
**3**	AOID	chr18: g.61323147T>C NM_006919.2 c.917A>G NP_008850.1 p.Asp306Gly NOVEL	NM_000624.5 c.175G>A NP_000615.3 p.Ala59Thr rs139961453 Allele freq = 0.0003658			

## Data Availability

Not applicable.
